# Individual differences in cognitive performance under pain linked to region-specific alpha power modulations

**DOI:** 10.1016/j.ynpai.2025.100196

**Published:** 2025-09-10

**Authors:** Francesca Storey, Mariya Prokhorenko, Michael L. Keaser, Patrick Skippen, Andrew J. Furman, David A. Seminowicz, Ali Mazaheri

**Affiliations:** a School of Psychology, University of Birmingham, United Kingdom; b Birmingham Medical School, University of Birmingham, United Kingdom; c Faculty of Life Sciences and Medicine, King's College London, United Kingdom; d Department of Neural and Pain Sciences, University of Maryland School of Dentistry, Baltimore, MD, USA; e Center to Advance Chronic Pain Research, University of Maryland Baltimore, Baltimore, MD, USA; f Institute for Genome Sciences, University of Maryland School of Medicine, Baltimore, MD, USA; g Data Sciences, Hunter Medical Research Institute, Australia; h School of Medicine and Public Health, University of Newcastle, Australia; i Department of Diagnostic Radiology and Nuclear Medicine, University of Maryland School of Medicine, Baltimore, MD, USA; j Department of Medical Biophysics, Schulich School of Medicine & Dentistry, University of Western Ontario, London, Ontario, Canada; k Centre for Human Brain Health, University of Birmingham, United Kingdom

**Keywords:** Pain, Cross-modal attention, Alpha oscillations, Individual variablity

## Abstract

Chronic pain is associated with reduced cognitive function, potentially due to a diversion of cognitive resources to processing pain. However, reduced cognitive function during pain is not always consistently evidenced, perhaps due to individual differences in the attentional cost of pain processing. In the current electroencephalography investigation, we therefore examined differences in the top-down modulation of oscillatory activity in a cognitive task, between participants who performed better, and worse, during experimentally induced neuropathic-like pain. We employed a cross-modal attention task in which visual cues indicated whether participants needed to judge the visual orientation or discriminate the auditory pitch of an upcoming target. The visual and auditory targets were presented either simultaneously or individually, enabling us to assess the “cost” of having a distractor present in each modality. Participants engaged in the task under two conditions: prolonged pain via the capsaicin-heat pain model, and pain-free. Participants less “costed” by visual distraction during pain demonstrated a greater increase in alpha power (8–12 Hz) over frontal/central electrodes during pain. This may reflect inhibition of regions related to the processing of painful stimuli (somatosensory cortex), which could increase availability of resources to meet task-demands – a possible task-favouring pattern. In conclusion, our results support the notion that better cognitive function during pain is associated with a behavioral strategy.

## Highlights

• Chronic pain is linked to reduced cognitive function, possibly due to attentional diversion to pain processing.
• Using EEG, we examined oscillatory activity modulation in a cross-modal attention task under pain and pain-free conditions.
• Participants who performed better during pain exhibited increased frontal/central alpha power (8–12 Hz).
• Increased alpha power may reflect inhibition of pain-processing regions, preserving cognitive resources for the task.
• Findings suggest individual differences in cognitive function under pain relate to variations in inhibitory control.

## Introduction

Chronic pain patients often report symptoms of altered cognition and ability to concentrate, colloquially known as ‘brain fog’ (Lucius, 2021). Meta-analytic data corroborate this, demonstrating reduced cognitive function in chronic pain patients compared to pain-free controls (Berryman et al., 2014, Bunk et al., 2019). Additionally, in the opposing direction, subjective pain sensitivity is reduced by engagement in a cognitive task (Bascour-Sandoval et al., 2019, Bunk et al., 2019), suggesting shared mechanisms underly pain and cognition (Domenichiello & Ramsden, 2019). Reduced cognitive function in patients may affect compliance with clinical guidance and ability to report pain, complicating therapeutic decision-making (Cole et al., 2006). Therefore, it is important to explore shared mechanisms to help guide chronic pain research and management.

The modulation of oscillatory activity in the alpha range (8–12 Hz), measured using EEG/MEG, has been proposed to play a role in ensuring proper allocation of resources to relevant brain regions by inhibiting sensory processing in task-irrelevant regions (Foxe and Snyder, 2011, Klimesch et al., 2007, van Diepen et al., 2019). This view is in line with the common observation that alpha power is increased in sensory regions associated with task-irrelevant processes (Hanslmayr et al., 2007, Lange et al., 2013, Romei et al., 2008a, Romei et al., 2008b, van Diepen and Mazaheri, 2017, van Dijk et al., 2008, Zumer et al., 2014,). Therefore, alpha power may be modulated to increase over irrelevant regions, closing the gates to processing in these regions and promoting the flow of information to relevant regions (Fu et al., 2001, Jensen and Mazaheri, 2010, Mazaheri et al., 2010 Klimesch et al., 2007).

Cognitive processes compete for a finite set of resources, and this resource diversion model is seen in both studies using fMRI and EEG in different populations (Addante et al., 2015, Cohen et al., 2015, Koen et al., 2018, Yeh and Koen, 2022). Pain may divert resources to areas of the brain processing painful stimuli, compromising resource availability for cognitive tasks. This may explain reduced cognitive function in chronic pain patients − a “chronic interruption by pain“ (Ecclestone & Crombez, 1999). However, others demonstrate pain does not always influence cognitive performance, suggesting pain may not have automatic priority to divert resources (González-Villar et al., 2017). Using fMRI, researchers have offered a possible explanation for the disparity. Participants who perform faster on a task during pain reveal task-related modulations in regions activated during painful stimulation, but these are not evident in participants responding slower during pain (Seminowicz et al., 2004, Erpelding and Davis, 2013, Cheng et al., 2017). This indicates pain’s effect on cognition depends upon an adopted strategy, prioritising pain or the cognitive task.

In the current investigation we employed a cross-modal attention task used in our previous investigations (Mazaheri et al., 2014, van Diepen and Mazaheri, 2017), whereby visual cues directed participants to either judge the visual orientation or discriminate the auditory pitch of an upcoming target. The visual and auditory targets were presented either simultaneously or individually, enabling us to measure the behavioral “cost” of having a distractor in each modality. Some cues (presented in unimodal trials) gave no information on the modality of the upcoming target, enabling us to measure the attentional “benefit” of informative cues. Our previous work has shown that preparing for visual discrimination (compared to auditory discrimination) led to a decrease in alpha power over occipital regions of the cortex (Mazaheri et al., 2014, van Diepen and Mazaheri, 2017).

Here, participants performed this task in two conditions: sustained pain, and no pain. We then compared pain-related modulations of alpha power during the task, between the following performance groups: participants with greater vs. lesser distraction cost during the condition of sustained pain; and between those with greater vs. lesser attentional benefit during pain. We aim to investigate whether pain’s effect on attentional performance is associated with a behavioral strategy, reflected by modulation of alpha power. We hypothesise participants with better attentional performance during pain, show less diversion of cognitive resources to pain-related areas, as seen by increased inhibitory alpha power over areas possibly related to processing painful stimuli and decreased alpha power over target-related areas.

## Methods

### Participants

44 pain-free neurotypical participants were recruited (22 males, mean age = 28.4, range 19–42 years), from the University of Maryland, Baltimore. 17 of these were assigned to a no-pain control condition. Resting state EEG data was collected for participants. The resting state EEG and 17 no-pain control participants’ data has been previously published addressing a different topic (Furman et al., 2018), and is not relevant to this study therefore not included. Task-related EEG data was collected from the remaining 27 participants and analyzed in this study.

Informed written consent was obtained from participants before study procedures and all data was anonymized. This study was approved by the University of Maryland, Baltimore Institutional Review Board and conducted in accordance with the Declaration of Helsinki (Furman et al., 2018).

### Materials

#### Model of capsaicin thermal heat pain

Participants’ heat pain thresholds (HPT; temperatures first perceived as painful) were determined in a sensory testing session. Four individual HPT measurements were collected, then averaged and rounded down to the nearest integer. 1 g of 10 % topical capsaicin cream (Professional Arts Pharmacy, Baltimore, MD) was applied to the volar surface of the left forearm, fixed in place with a Tegaderm bandage. A 30 x 30 mm Medoc pathway ATS Peltier thermode (32 °C) was applied on top (Medoc Advanced Medical Systems Ltd., Ramat Yishai, Israel). Pain intensity ratings were collected every 3 min for 15 min. The temperature was then increased to 3 °C below the HPT. For 5 min, pain ratings were provided every minute; if no pain was reported, the temperature was adjusted in 1 °C increments, but never exceeding HPT-1 °C (Furman et al., 2018).

#### Cross-modal attention task

**Fig. 1:**
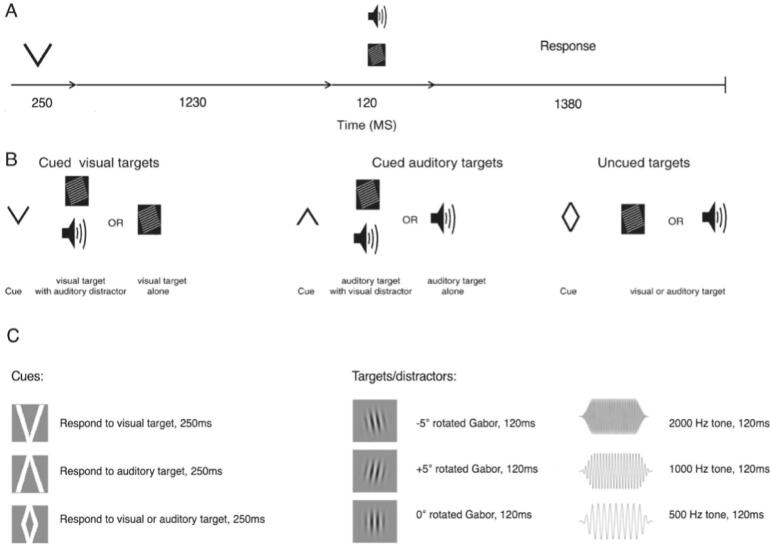
Cross-modal attention task showing timings (A), trial types (B) and stimuli used (C). Cues were displayed for 250 ms followed by a blank screen for 1230 ms, then a target for 120 ms. Participants had a further 1380 ms to respond (maximum response time 1500 ms). A downwards arrow signaled for a visual target, presented alone, or with a simultaneous auditory distractor, and an upwards arrow, for an auditory target (alone or with a visual distractor). A “rhombus-shaped” cue indicated participants should respond to an upcoming visual or auditory stimulus presented alone. Visual stimuli consisted of Gabor patches rotated to different degrees. Auditory stimuli consisted of tones played at three pitches. This image has been adapted from Mazaheri et al. (2014) and van Diepen et al. (2015).

The task was adapted from Mazaheri et al. (2014). A cue was displayed (250 ms) for an upcoming target (see Fig. 1). Participants were given instruction that a downwards arrow indicated to make a judgement on the degree of rotation of an upcoming visual target, either presented alone, or with a simultaneous auditory distractor, and an upwards arrow, to make a pitch judgment on an auditory target, again presented alone or with a visual distractor (see Fig. 1C for a description of stimuli). In 20 % of trials, a “rhombus-shaped” cue was presented, representing an uninformative cue, whereby participants should respond to either a visual or auditory target, presented alone with no distractor. Targets and distractors were displayed 1480 ms after cue onset, for 120 ms. Participants had a maximum of 1500 ms to respond, pressing a labelled button on a response box. Participants completed 300 trials (120 auditory, 120 visual, 60 ambiguous) randomized within five blocks for both conditions (a total of 600 trials). At the end of each block, participants paused to rate pain intensities (see Furman et al., 2018, for analysis of pain intensities).

#### EEG data acquisition

EEG was recorded using a 64-channel Brain Vision actiCAP system (Brain Products GmbH, Munich, Germany). Electrodes were placed using the international 10–20 system (Oostenveld & Praamstra, 2001), and impendences were maintained below 5 kΩ. Activity was recorded with a sampling rate of 100 Hz and within a 0.01–100 Hz bypass filter. A BrainAmp DC amplifier (Brain Products GmbH, Munich, Germany) was used to amplify the signal, which was digitalized using Brain Vision Recorder software (v. 2.1, Brain Products GmbH, Munich, Germany; Furman et al., 2018). The EEG data was referenced online to an electrode placed on the right earlobe with a common ground set at the FPz site.

### Procedure

Participants had the EEG apparatus set up, were trained on, then performed the task with a 32 °C non-painful thermode. Participants then underwent capsaicin incubation and completed the task again with ongoing capsaicin thermal heat pain.

### Data analysis

#### Behavioral data

A two-way Analysis of Variance (ANOVA) in IBM SPSS Statistics (V. 27.0), tested for the effects of independent variables, condition (no pain vs. pain) and target modality (auditory vs. visual), on the dependent variable, reaction time. A three-way ANOVA was utilised to test for effects and interactions of the independent variables, distractor presence (unimodal vs. bimodal), condition and target modality, on reaction time. This was repeated for cue type (unimodal trials with informative cues vs. unimodal trials with uninformative cues), condition and target modality.

#### Performance grouping

Distraction cost was calculated for individual participants by subtracting mean reaction times in unimodal trials from bimodal trials, for each pain state and target modality type. Participants were then sorted into the following performance groups based on their distraction cost, separately for each modality:

1. Participants with a greater distraction cost during the painful condition, than no-pain
2. Participants with a lesser distraction cost during the painful condition, than no-pain

Attentional benefit was calculated for individual participants by subtracting mean reaction times in unimodal trials with informative cues from those with uninformative cues, for each pain state and target modality type. Participants were then sorted into a further two groups based on their attentional benefit, separately, for each modality:

3. Participants with a greater attentional benefit during the painful condition, than no-pain
4. Participants with a lesser attentional benefit during the painful condition, than no-pain

#### EEG processing

The preprocessing of the EEG data was conducted using EEGLAB (Delorme & Makeig, 2004) and Fieldtrip toolbox (Oostenveld et al., 2011). The data was referenced offline to the average of all of the channels. To extract relevant time intervals, data was time-locked to cue onset and epoched from −1 to 3.5 s. Ocular artefacts were removed based on the scalp distribution using independent component analysis (“runica”) in EEGLAB. The mean number of removed components was 1.59 out of 30 for each participant (total 43 components removed from 810 for all participants; 5.3 % of the data; see Supplementary Material, Table S1).

#### Frequency analysis

Grand average time–frequency representations of power were calculated using a Hanning taper for each factor of interest (target modality, condition of pain, distraction cost and attentional benefit groups), to allow for examination of the course of alpha activity averaged over participants and trials (see supplementary material, Fig. S1). A sliding time window with steps of 50 ms was tapered with a frequency-dependent Hann window length, of three cycles for each frequency (Δ*T* = 3/*f*), in line with Mazaheri et al., 2010, Mazaheri et al., 2014, Mazaheri et al., 2009 and van Diepen and Mazaheri (2017). The time frequency data was normalized as the relative change = (Power−Baseline Power [−0.5 to 0.1 s before cue])/Baseline Power).

The time–frequency representations during no pain were then subtracted from those during pain, for each target modality, distraction cost and attentional benefit groups, to allow for examination of possible pain-related oscillatory activity.

### *Statistical analysis*

We conducted the following comparisons:

1. Changes in oscillatory activity induced by visual versus auditory cues across all participants, collapsed across pain/no-pain conditions.
2. Changes in pain-related oscillatory activity between participants who exhibited a greater distraction cost during pain with those who exhibited a lesser distraction cost during pain.
3. Changes in pain-related oscillatory activity in those with a greater attentional benefit during pain with those who had a lesser attentional benefit during pain.

For each comparison, we implemented a cluster-based permutation technique as described by Maris and Oostenveld (2007) to address multiple comparisons issues. This method involved a dependent samples *t*-test for target modality contrasts (auditory vs. visual) and independent samples t-tests for group contrasts (greater vs. lesser distraction cost during pain; greater vs. lesser attentional benefit during pain), for every point in the electrode-by-time plane from 0.5 s before cue onset to 3 s after. T-statistics at adjacent spatiotemporal points were clustered if they exceeded a threshold of *p* < 0.05 (cluster alpha). The probability value for each cluster was determined using a Monte Carlo estimate of the permutation *p*-value, obtained by randomly swapping conditions among participants 1000 times and calculating the maximum cluster-level test statistic.

Bayes factors (BF_10_; H_0_/H_A_) were then calculated for non-significant comparisons across time points in which significant clusters arose, using independent samples Bayesian t-testing, computed using JASP (V.0.19.1). Wagenmakers et al. (2017) interpretation was utilised: BF_10_ between 1 and 3 yields anecdotal evidence for H_A_; between 1 and 0.33 presents anecdotal evidence for H_A_ (1:3 probability in favour of H_0_); between 0.33 and 0.10 presents moderate evidence for H_0_, and <0.10 strong evidence for H_0_.

## Results

### Behavioral results

#### Modality difference

Participants responded faster in visual, rather than auditory discrimination trials (699.30 ms vs. 704.23 ms; F(1, 26) = 286.99, p < 0.001; see Fig. 2.1).

**Fig. 2:**
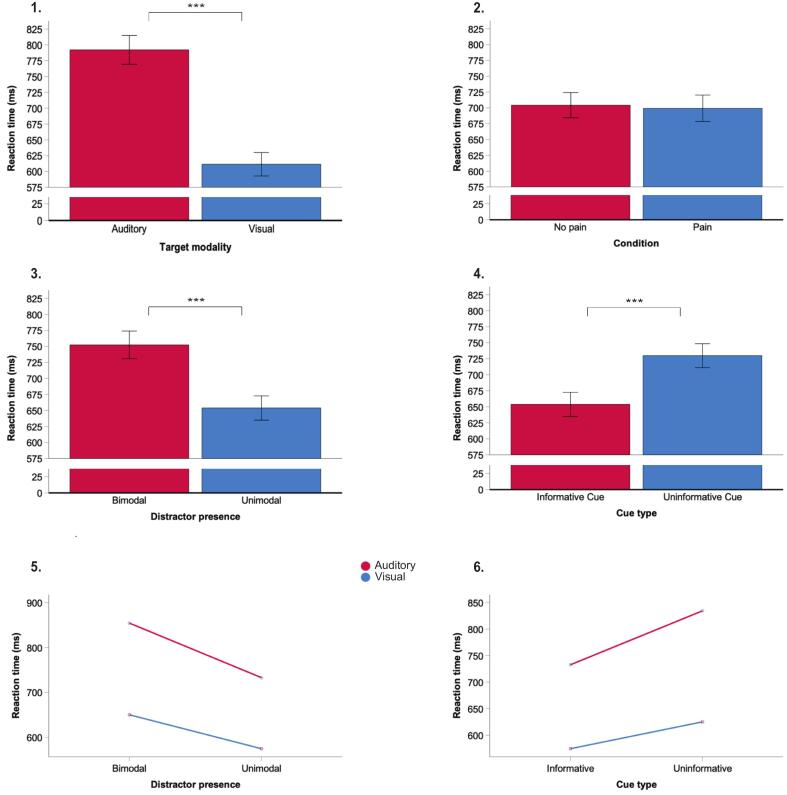
Reaction time as a function of target modality (1), condition (2), distractor presence (3) and cue type (4), and interaction plots for significant interactions (5, 6). Participants were significantly faster in trials with auditory than visual targets (1), participants’ response times were similar between no pain and pain conditions (2), participants responded significantly faster in unimodal (distractor absent) than bimodal (distractor present) trials (3), participants responded significantly faster after being presented with informative rather than uninformative cues (4). There was a significant interaction between distractor presence and target modality, whereby typical effects of distractor presence were enhanced for auditory discrimination trials (5). There was also a significant interaction between cue type and target modality, whereby typical effects of cue type were enhanced for auditory discrimination trials (6). Asterisks (***) denote statistical significance (*p* < 0.05). Error bars denote standard error (+/−1).

#### Pain state

Participants performed similarly across pain and no-pain conditions (F(1, 26) = 0.39, p = 0.539; see Fig. 2.2. Additionally, there were no interactions between modality and condition, suggesting effects of modality were consistent across conditions.

#### Distractor presence

Participants responded slower in bimodal (distractor present) compared to unimodal (distractor absent) trials (752.28 ms vs. 653.80 ms, F(1, 26) = 145.19, p < 0.001; see Fig. 2.3). There was a significant interaction between distractor presence and target modality (F(1, 1) = 9.27, p = 0.005), whereby typical effects of distractor presence were enhanced for auditory discrimination trials (see Fig. 2.5).

#### Cue type

Participants responded faster in trials where informative cues were presented in comparison to trials where uninformative cues were presented (653.80 vs. 729.90, F(1, 26) = 143.58, p < 0.001; see Fig. 2.4). There was also a significant interaction between cue type and target modality (F(1, 1) = 21.06, p < 0.001), whereby typical effects of cue type were enhanced for auditory discrimination trials (see Fig. 2.6). Variability has been displayed utilizing scatterplots (view Supplementary Material Fig. S2).

### EEG data

#### Modality specific alpha/beta modulation as a result of cross-modal attentional cues

**Fig. 3:**
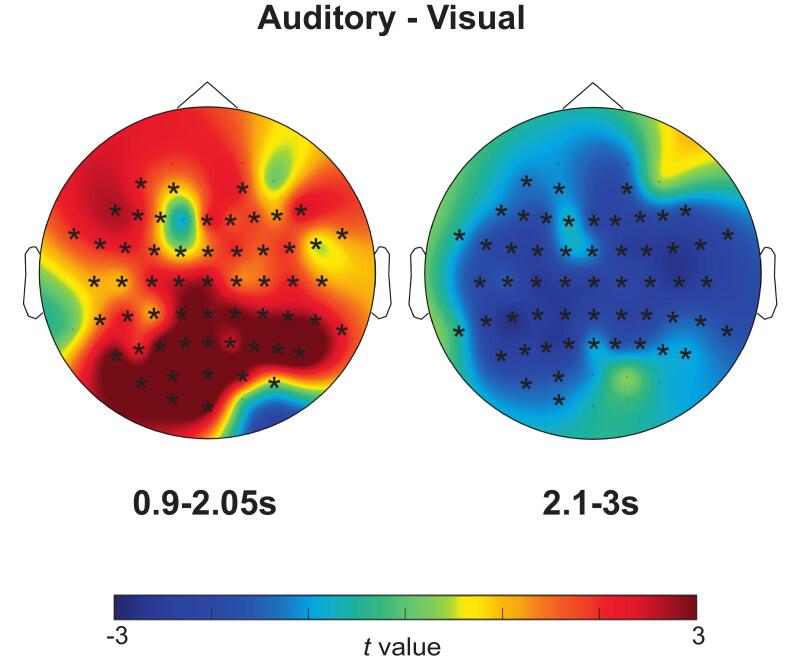
Scalp topographies showing mean difference in alpha power between trials with auditory and visual targets (auditory − visual). The colour bar denotes the t value distribution. Data is shown averaged over frequency (8–12 Hz). In trials with auditory targets compared to visual targets, alpha power was higher from 0.9 to 2.05 s but lower from 2.1 to 3 s. Asterisks (*) represent electrodes that showed a significant difference between these times (p < 0.05).

We found alpha power was increased over posterior to central electrodes during preparation for auditory compared to visual discrimination. Specifically, the contrast between cues signaling auditory versus visual targets revealed a significant difference in alpha power that was most pronounced between 0.9 to 2.05 ms (*p* < 0.001, t = 2392.15; see Fig. 3A*)* starting posteriorly, spreading centrally. In addition, alpha power was decreased generally after target display for auditory compared to visual discrimination, specifically the difference was most pronounced between 2.1 and 3 s, *p* < 0.001, t = 1482.16 (see Fig. 3B).

#### Participants with less visual distraction cost while in pain had a central alpha increase

**Fig. 4:**
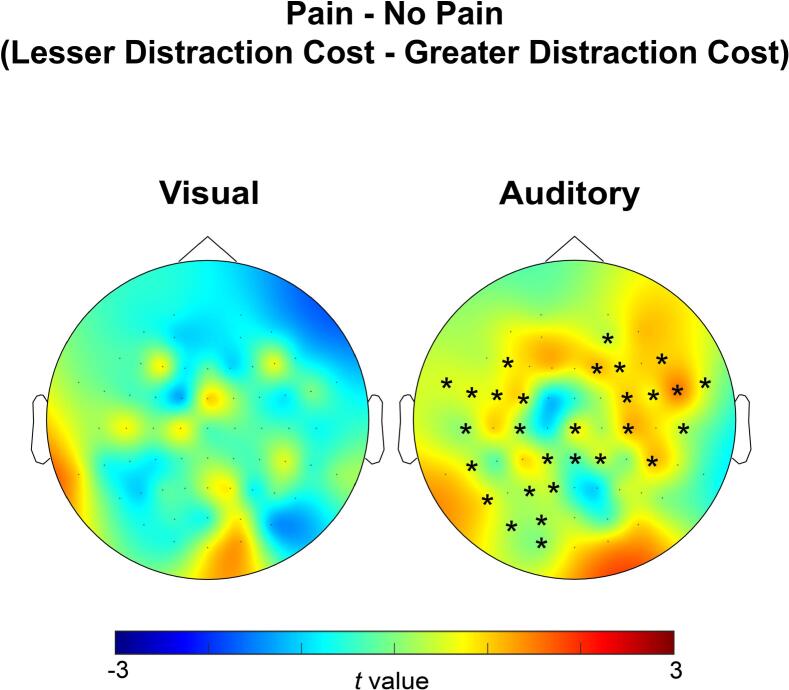
Scalp topographies exhibiting the difference in pain-related alpha power (pain − no pain) between participants who were more and less costed by distractors during pain (lesser distraction cost during pain − greater distraction cost during pain), during visual (left) and auditory (right) discrimination. The colour bar denotes the t value distribution. Topographies are shown averaged over frequency (8–12 Hz). Asterisks (*) represent electrodes that showed a significant difference (p < 0.05). Participants less costed by distractors during pain had a central alpha power increase in pain, relative to no-pain, during auditory discrimination that started anteriorly and spread posteriorly, peaking from 2 to 2.1 s.

We found that participants with a lower distraction cost during pain had significantly higher alpha power during the pain condition (relative to no pain) during auditory discrimination, peaking at 2 to 2.1 s over a cluster of central electrodes, compared to participants with a higher distraction cost during pain, *p* = 0.042, t = 150.38 (see Fig. 4 for the distribution of electrodes in the cluster). This cluster started anteriorly and spread posteriorly.

During visual discrimination, no significant pain-related clusters were identified between distraction cost groups. As Cz had emerged in previous contrasts, it was included as an electrode of interest. For this comparison, Bayesian independent samples *t*-test revealed BF_10_ = 0.816 (2–2.1 s; electrode Cz), in the direction of a greater alpha power in participants with a greater distraction cost during pain. According to Wagenmakers et al. (2017) interpretation, this Bayes Factor provides anecdotal evidence in support of the null hypothesis (no difference between groups).

In addition, alpha power was compared between these performance groups (lesser distraction cost during pain > greater distraction cost during pain) separately for each condition (pain, and no pain), however no significant clusters were identified (see supplementary material, Fig. S3).

#### There were no pain-related alpha changes between participants with a greater vs lesser attentional benefit during pain

The same analyses were performed between participants with greater and lesser attentional benefit in pain. No significant clusters arose between groups. For auditory discrimination trials, Bayesian independent samples *t*-test (Cz; 2 to 2.1 s) revealed BF_10_ = 0.473, in the direction of a greater alpha power in the participants with less attentional benefit during pain. For visual discrimination trials, BF_10_ = 0.669, in the direction of greater alpha power in the group with a greater attentional benefit during pain. Bayesian statistics for both target modalities indicate anecdotal evidence in support of the null hypothesis.

## Discussion

This study aimed to investigate if pain’s effect on attentional performance can be associated with a behavioral strategy, reflected by alpha power modulation. Taken overall, we found participants better at ignoring visual distractors during auditory discrimination trials, when they are in pain, may adopt a strategy to ignore distracting input. This is reflected by increased alpha power over central electrodes, possibly overlying areas of pain-related activity. We however, found no evidence for alpha power variation between those with a greater and lesser attentional benefit from informative cues during pain.

On general inspection of the data, alpha power was increased over posterior electrodes during preparation for auditory compared to visual targets. This suggests greater inhibition over the occipital cortex preparing for auditory discrimination, closing the gates to areas associated with distracting input from visual stimuli, in line with existing literature using this cross-modal attention paradigm (Mazaheri et al., 2014, van Diepen and Mazaheri, 2017, van Diepen et al., 2015). Additionally, we noted a widespread decrease in alpha power after target display for auditory compared to visual discrimination. This decrease in inhibition, suggests a requirement for greater cognitive resource allocation globally, suggesting processing auditory targets may have been more demanding for participants. Behavioral data was in line with this. Participants were slower to respond to trials with auditory targets. In addition, participants were slower to respond to trials with distractors as expected, but, even more so during auditory discrimination. This implies distractors had a greater cost to participants’ performance during auditory discrimination, possibly suggesting participants had less control over reactive attention systems during auditory discrimination. As expected, participants responded to targets quicker when cued with informative information on upcoming target modality. However, this effect was exaggerated during auditory discrimination. This could suggest participants relied more heavily on the attentional benefit they received from informative cues during these trials, implying greater need to engage proactive attention systems for auditory discrimination. Taken together, we therefore speculate auditory discrimination during this task was more challenging for participants.

After grouping participants based on their performance during pain, we found participants less costed by distractors during pain displayed a centrally located increase in alpha power in the painful (relative to not painful) condition. This may be reflective of increased inhibition over the somatosensory cortices, which are activated during painful stimulation. Increased inhibition here may facilitate increased flow of cognitive resources to regions processing targets, as participants focus on targets and ignore distraction. This interpretation corroborates with the fMRI study by Seminowicz et al. (2004), as they observed reduced activity in the primary and secondary somatosensory cortices, and the anterior insula during engagement in a cognitive task, in only those who responded faster during pain, supporting a ‘pro-task’ strategy. In addition to this, existing literature has shown corresponding reductions in the perception of pain intensity in relation to reductions in pain-related brain activity during focused attention on a task (Bantick et al., 2002). So, it may be of interest to expand our research and look for associations between perceived pain intensity and our pattern of alpha power modulation, to investigate whether this strategy could be not only, ‘pro-task’, but also, ‘anti-pain.’

This pattern was only noted for trials with auditory, not visual targets. As discussed, our data suggests auditory trials may have been more challenging for participants. In addition, others have demonstrated a significant reduction in pain-related activity in more challenging trials (Bantick et al., 2002), so, there appears to be a relationship between task difficulty and modulation of pain-related activity. Thus, perhaps, visual discrimination trials were not demanding enough to evoke this strategy. It may be the case that only in times of true, or threatened, resource compromise, mechanisms to inhibit input from competing painful stimuli are called upon. Considering this, in the opposing direction, task-positive fMRI networks are activated with increasing pain intensity (Seminowicz & Davis, 2007), suggesting stronger compensatory neural responses are evoked at higher pain intensities to maintain cognitive performance. So, it may be possible that pain may not have been intense enough to compromise resources and evoke strategies of alpha modulation in easier trials with visual targets.

In contrast to our hypotheses, we did not observe a corresponding lower alpha power over target-related areas in this group. This was surprising, as lower alpha power over target-related areas would suggest a greater allocation of resources to these regions and has been associated with faster responses in the same cross-modal attention task (Mazaheri et al., 2014). However, the target-related area for auditory discrimination trials would be the auditory cortex. We cannot dismiss the possibility of modulatory activity here. As from previous research, we know changes in alpha activity over the auditory cortex are difficult to exhibit using EEG, due to its size and anatomical location (Bastiaansen and Brunia, 2001, Mazaheri et al., 2014, van Diepen et al., 2015). Therefore, it is difficult to assess using EEG whether increased inhibition over areas possibly related to pain was associated with a diversion of cognitive resources to auditory discrimination areas.

No significant differences in condition-related modulation of alpha power were found between participants who benefitted more or less from the attentional cues during pain. This suggests variation of proactive attention in pain is not explained by modulations of detectable alpha. Alternatively, deep structures involved in pain and attention, that are difficult to detect using EEG, may have been modulated between groups. An example could be the anterior insula, located deep within the lateral sulcus. In the same fMRI study by Seminowicz et al. (2004), pain-related activity in the anterior insula was attenuated by engagement in a cognitive task in only participants who performed better in the task. Thus, it may be possible that those who more proactively used the cues for their attentional benefit during pain show increased inhibitory alpha power over the anterior insula; however, we could not capture this with extracranial EEG.

Reasonable next steps would include to investigate for this strategy in chronic pain patients with and without cognitive disruption, to assess clinical relevance of our results. In addition, cognitive disturbance is observed in a range of conditions, where pain may not be present or the most troublesome symptom, including pregnancy, the post-partum period, sleep deprivation, insomnia, diabetes and after chemotherapy (Conti et al., 2024, Olaithe et al., 2018, Xu et al., 2020, Younis et al., 2025, Zhang et al., 2020). Thus, it would be useful to conduct similar comparisons to see if patients without cognitive disruption exhibit strategies of alpha modulation that those with cognitive disruption do not, to see if our results are pain-specific or perhaps more likely related to physical stress as a whole.

Subsequent research should explore if this strategy can be trained or developed, aiming to improve focus during pain. Cognitive behavioural therapy has been associated with improvements in inattention symptoms in patients with ADHD (Liu et al., 2023). In addition, theta entrainment techniques, such as, audiovisual stimulation, and transcranial electrical stimulation have been associated with improved cognitive performance (Addante et al., 2021, Boudewyn et al., 2018, Boudewyn et al., 2020, Roberts et al., 2018, Hanslmayr et al., 2019, Hoy et al., 2013, Mizrak et al., 2018). Furthermore, a relationship between alpha and theta also may play a role in focusing attention (Mazaheri et al., 2010, Mazaheri et al., 2009). Thus, it may be of interest to explore these interventions to see if their effects extend to attention during pain, and alpha.

In conclusion, our results provide evidence that the ability to maintain deeper focus and avoid reactively attending to distractors during sustained pain may be strategy-dependent, as reflected by differences in alpha oscillatory activity. This is a step towards understanding the link between cognitive dysfunction – or brain fog – and chronic pain.

## CRediT authorship contribution statement

**Francesca Storey:** Writing – original draft, Formal analysis. **Mariya Prokhorenko:** Data curation. **Michael L. Keaser:** Methodology, Formal analysis, Conceptualization. **Patrick Skippen:** Formal analysis. **Andrew J. Furman:** Formal analysis, Data curation, Conceptualization. **David A. Seminowicz:** Writing – review & editing, Funding acquisition, Formal analysis, Conceptualization. **Ali Mazaheri:** Writing – review & editing, Methodology, Funding acquisition, Formal analysis, Data curation, Conceptualization.

## Declaration of competing interest

The authors declare that they have no known competing financial interests or personal relationships that could have appeared to influence the work reported in this paper.

This work was supported by an IASP Collaborative Research Grant awarded to DS and AM. AM was supported by the Wellcome Leap ‘Untangling Addiction’ programme at the time of writing.

## Data Availability

The authors do not have permission to share data.
