# Supplementary material for: Individual differences in cognitive performance under pain linked to region-specific alpha power modulations
Source: Neurobiol Pain. 2025 Sep 10;18:100196. doi: 10.1016/j.ynpai.2025.100196 (PMC12550171; doi:10.1016/j.ynpai.2025.100196)
Supplement: Supplementary Data 1 [file mmc1.pdf]

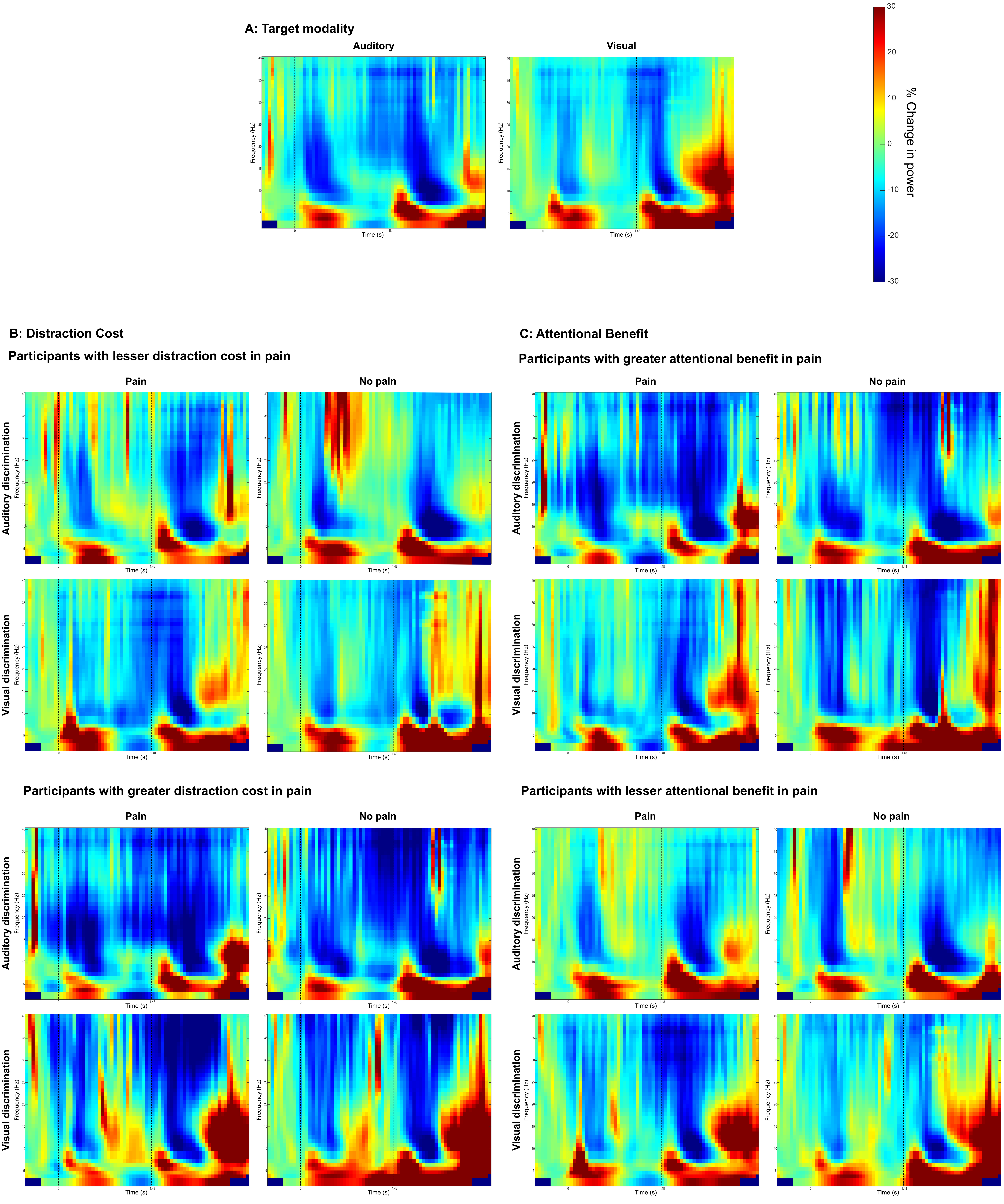

Figure S1. Time frequency representations of relative change in power over electrodes, for each factor of interest. Power is shown relative to baseline (-0.5s to -0.1s) averaged over participants, trials and electrodes. The colour bar denotes the % change in power relative to baseline. Time is shown on the x axis. Cue onset was at 0s and target onset was 1.48s. Frequency (Hz) is shown on the y axis. The top (A) time frequency representations show the course of oscillatory activoty for auditory and visual target modalities, collapsed across condition of pain. The lower time frequency representations show the course of oscillatory activity for distraction cost (B) and attentional benefit groups (C) for each target modality and condition of pain.
