# Supplementary material for: Individual differences in cognitive performance under pain linked to region-specific alpha power modulations
Source: Neurobiol Pain. 2025 Sep 10;18:100196. doi: 10.1016/j.ynpai.2025.100196 (PMC12550171; doi:10.1016/j.ynpai.2025.100196)
Supplement: Supplementary Data 2 [file mmc2.pdf]

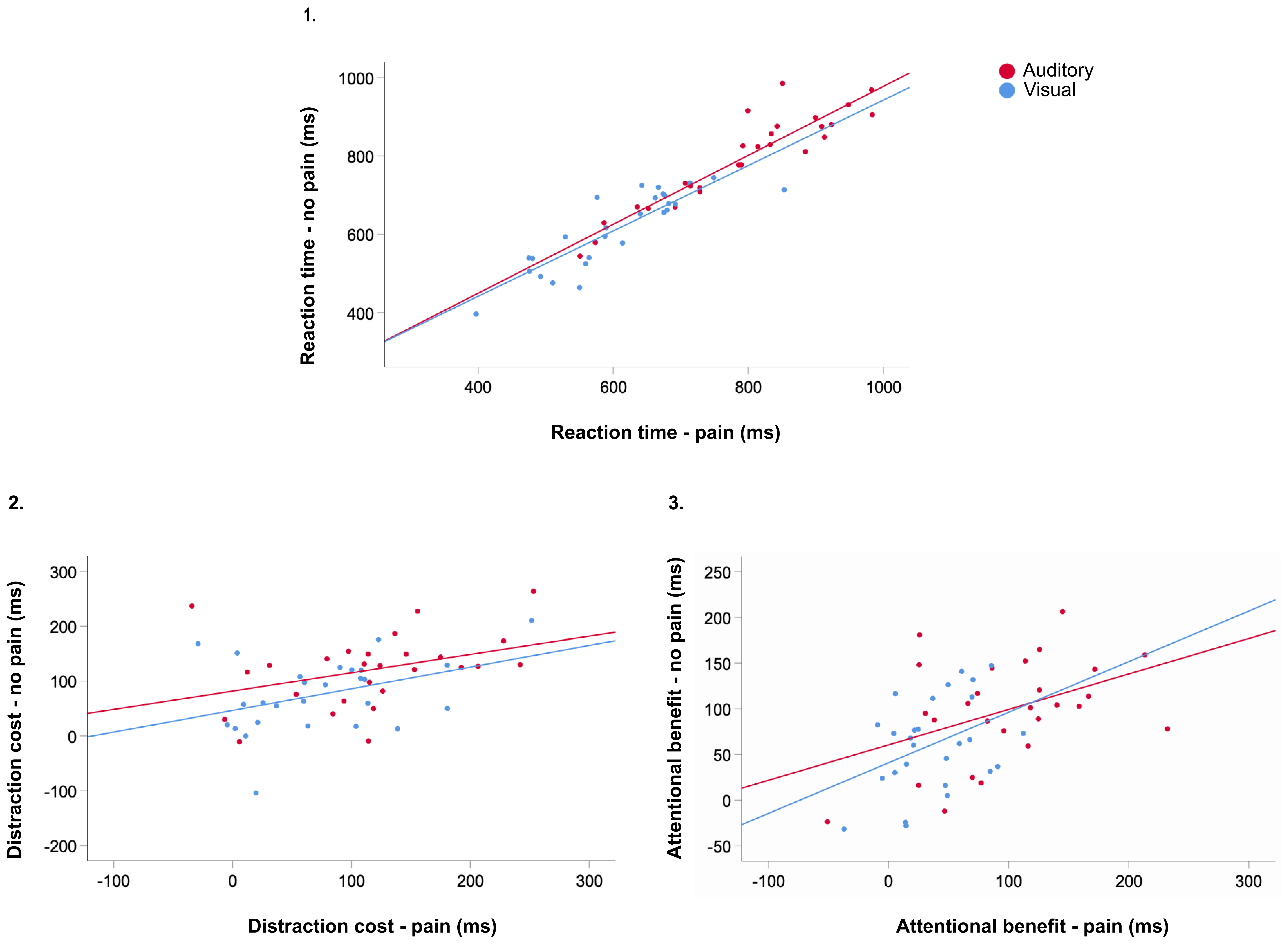

Figure S2. Scatterplots of individual reaction time, distraction cost and attentional benefit. Each data point represents mean participant overall reaction time (1), distraction cost (2) and attentional benefit (3), under the pain condition (x-axis) plotted against the no pain condition (y-axis). Data is presented separately for trials with auditory (red markers) and visual (blue markers) targets. Likewise, best-fit lines are shown independently for auditory (red) and visual (blue) target modalities. Distraction cost (2) was calculated by subtracting the reaction time in unimodal trials (distractor absent) from bimodal trials (distractors present). Attentional benefit (3) was calculated by subtracting reaction times in trials with informative cues from those with uninformative cues.
