# Supplementary material for: Individual differences in cognitive performance under pain linked to region-specific alpha power modulations
Source: Neurobiol Pain. 2025 Sep 10;18:100196. doi: 10.1016/j.ynpai.2025.100196 (PMC12550171; doi:10.1016/j.ynpai.2025.100196)
Supplement: Supplementary Data 3 [file mmc3.pdf]

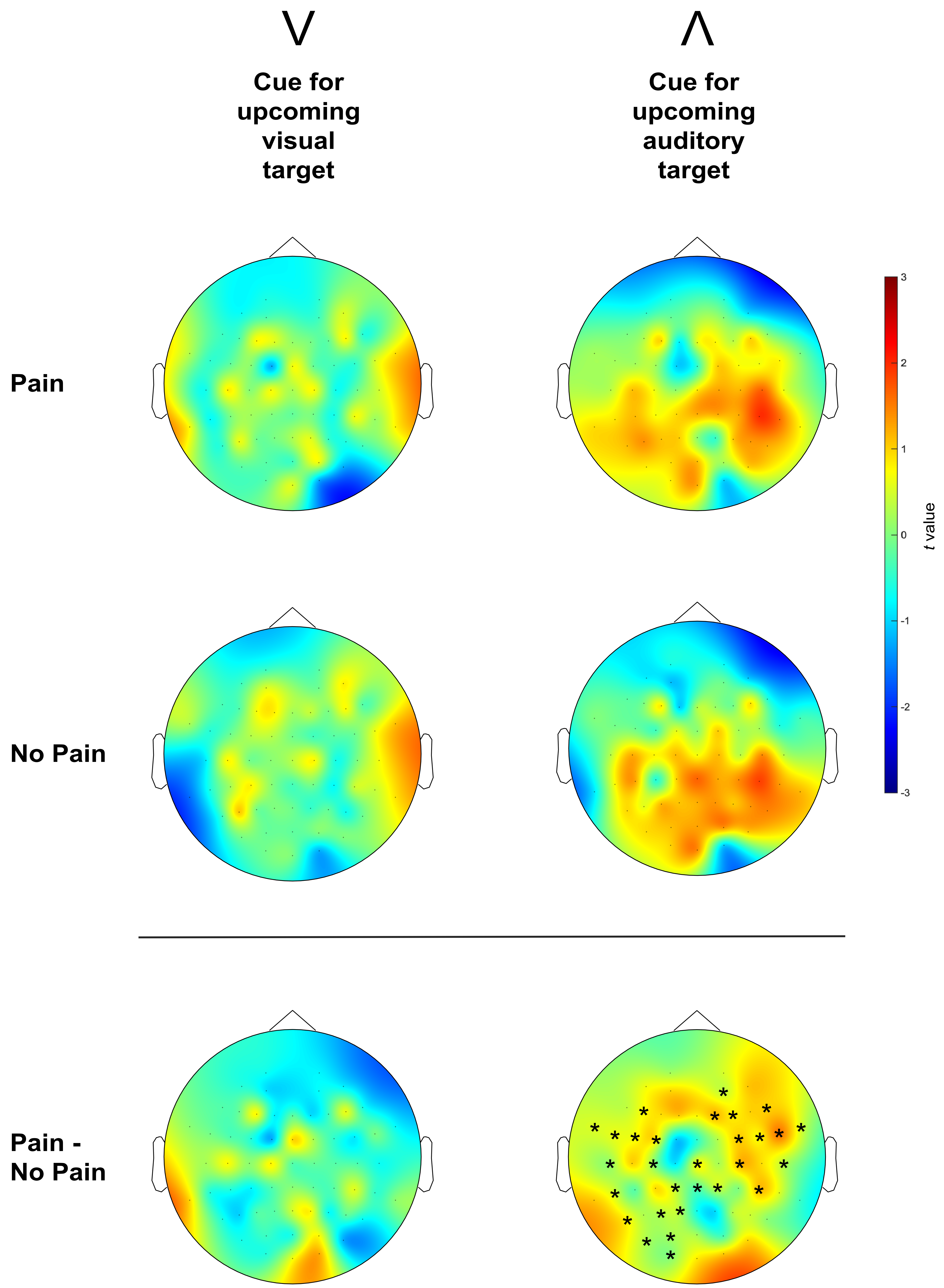

Figure S3. Scalp topographies exhibiting the difference in alpha power between participants who were more and less costed by distractors during pain (lesser distraction cost during pain > greater distraction cost during pain).The colour bar denotes the *t* value distribution. Data is shown averaged over frequency (8-12Hz). Asterisks (*) represent electrodes that showed a significant difference ($p < .05$). No significant differences were revealed when average alpha power for participants with a greater distraction cost in pain, was compared to participants with a lesser distraction cost in pain, separately for the pain (top row), and no pain (second row) conditions. However, participants less costed by distractors during pain, had a central alpha power increase in pain, relative to no pain (pain - no pain), during auditory discrimination, that started anteriorly and spread posteriorly, peaking from 2 to 2.1s (bottom row).
