# Supplementary material for: Individual differences in cognitive performance under pain linked to region-specific alpha power modulations
Source: Neurobiol Pain. 2025 Sep 10;18:100196. doi: 10.1016/j.ynpai.2025.100196 (PMC12550171; doi:10.1016/j.ynpai.2025.100196)
Supplement: Supplementary Data 4 [file mmc4.pdf]

Valid trials after artifact rejection	Distraction Cost (DC)				Attentional Benefit (AB)			
	DC in pain < no pain		DC in no pain < pain		AB in pain < no pain		AB in no pain < pain	
	Auditory Target	Visual Target	Auditory Target	Visual Target	Auditory Target	Visual Target	Auditory Target	Visual Target
Mean	28.29	28.56	28.54	28.18	28.5	28.56	28.33	28.11
Median	28	29	29	28	28.5	29	28	28
Range	28-29	28-29	27-29	27-29	28-29	28-29	27-29	27-29

Table S1. Mean, median, and range of valid trials after artifact rejection, separated by performance group (distraction cost, attentional benefit) and target modality types (auditory, visual). Artefacts were removed based on the scalp distribution using independent component analysis (“runica”) in EEGLAB.
